# Complete mitochondrial genome of the gray-headed lapwing (*Vanellus cinereus*) from Ningxia Hui Autonomous Region, China

**DOI:** 10.1080/23802359.2021.1882911

**Published:** 2021-03-01

**Authors:** Hao Zhai, Dehuai Meng, Yuhui Si, Zongzhi Li, Zeyan Cui, Hongxian Yu, Liwei Teng, Zhensheng Liu

**Affiliations:** a College of Wildlife And Protected Area, Northeast Forestry University, Harbin, PR China; b Key Laboratory of Conservation Biology, National Forestry And Grassland Administration, Harbin, PR China; c State-Owned Xinbin Manchu Autonomous County Tonggou Forest Farm, Fushun, PR China

**Keywords:** Complete mitochondrial genome, *Vanellus cinereus*, Charadriidae

## Abstract

We determined the whole mtDNA genome of the gray-headed lapwing (*Vanellus cinereus*) in Ningxia Hui Autonomous Region, China. The complete mitochondrial genome is 17,078 bp in length and consists of 13 protein-coding genes (PCGs), 22 tRNA genes, 2 rRNA genes, and 1 control region (D-loop). The nucleotide composition is 31.65% A, 23.50% T, 13.76% G, and 31.09% C. The result of phylogenetic analysis showed that there was close genetic relationship between *V*. *cinereus* and *V. vanellus*.

The genus *Vanellus* is composed of 22 species of large plovers or lapwings (Perrins and Middleton 1984), several of which commonly breed in agricultural areas (Sonobe and Usui 1993). The gray-headed lapwing (*Vanellus cinereus*) is a charadriid bird belonging to the Order Charadriiformes, which breeds in northeast China and Japan (She et al. 2016).

We sequenced the mitochondrial genome of *V*. *cinereus*. Our samples were obtained from fresh muscle of the *V. cinereus* from natural death in the Ningxia Hui Autonomous Region, China (105°57′E, 37°44′N). These specimens were stored in College of Wildlife and Protected Area, Northeast Forestry University (No. HTMJ2020). DNA library was constructed using MGIEasy DNA Library Prep Kit (MGI, China) and sequenced by MGI MGISEQ-2000 with 150 paired-ends. The annotation and phylogenetic tree of the mitogenome sequence were conducted by MITOS (Bernt et al. 2013) and MEGA version 7 , respectively.

The complete mitochondrial genome of *V*. *cinereus* was 17,078 base pairs (bp) in length and consisted of 37 genes, including13 protein-coding genes (PCGs), 2 rRNA genes (12S ribosomal RNA and 16S ribosomal RNA), 22 tRNA genes, and 1 control region (D-loop). The nucleotide composition is 31.65% A, 23.50% T, 13.76% G, and 31.09% C. The total length of 13 PCGs is 11,397 bp in length, all of which are encoded on the same strand except for ND6 in the heavy strand (H strand). In 13 PCGs, except ND1 begins with ATA, ND3 begins with ATC, COX1, and ND5 begins with GTG, the remaining nine PCGs beginning with ATG (*CYTB*, *ND2*, *ND6*, *ATP6*, *ATP8*, *COX2*, *COX3*, *ND4L*, and *ND4*) as the start codon. The total length of 22 tRNA genes is 1550 bp in length, and ranges from 66 to 74 bp are interspersed along the whole genome. The sequence lengths of the 12s RNA and 16s RNA genes are 975 and 1600 bp, respectively, and that of the D-loop region (control regions) is 1562 bp. All these information and assembled sequence were submitted to GenBank with accession number MW303998. In addition, the raw sequencing data were deposited in SRA (SRA no. PRJNA686450).

The phylogenetic relationship was inferred by using the maximum likelihood method based on the Tamura–Nei model (Tamura and Nei 1993) and conducted in MEGA7 (Kumar et al. 2016) . The bootstrap consensus tree inferred from 1000 replicates is taken to represent the evolutionary history of the taxa analyzed (Felsenstein 1985). This affirmed the monophyly of the two *Vanellus* species included in the analysis (Figure 1).

**Figure 1.**
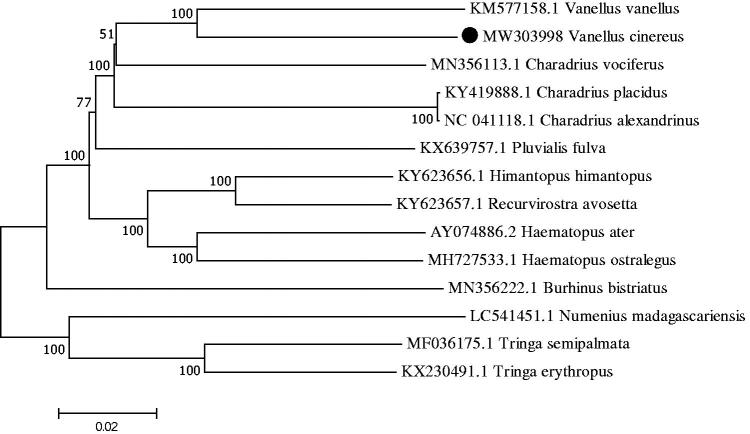
Phylogenetic tree generated using the maximum likelihood method based on complete mitochondrial genomes of 14 species.

## Data Availability

The data that support the findings of this study are openly available in GenBank of NCBI at https://www.ncbi.nlm.nih.gov/, reference number MW303998. The associated SRA number is PRJNA686450.
